# Types of Pulmonary Tuberculosis Wherein Only Much Granules Occur in the Sputum

**Published:** 1916-03

**Authors:** 


**Types of Pulmonary Tuberculosis Wherein Only Much Granules Occur in the Sputum.** Ralph C. Matson, Portland, Ore., N. M. Med. Jour., Dec. Much is a proper name. Quite a thorough bibliographic history of the condition is given. The characteristic pathology of these cases is the proliferation of tuberculous tissue and its transition into fibrous tissue with little tendency to softening. Thus they correspond fairly well with the old conception of fibroid phthisis. Secondary contraction is a marked feature, followed by compensatory emphysema with asthmatic symptoms. Unless from exacerbations, there is no fever and the course is usually slow and without cachexia. Retraction and narrowing of the intercostal spaces occurs, with retarded motion or fixation. Fluoroscopic examination shows the diseased side smaller and darker, with ascent of diaphragm and displacement of larynx and trachea toward the diseased side. Physical methods alone are not, however, definitely diagnostic. D'Espine's sign, noted by Stoll has not been confirmed by the author. The tuberculin test is always negative. Tubercle bacilli are few if any and are usually detected only by concentration methods, of which preference is given to Ellermann and Erlandsen's. Of 164 suspected cases which did not show bacilli by direct methods, 14 revealed them by the Uhlenhuth method, 22 by the Sculte method, 36 (including the foregoing fractions of the series) by the Ellerman and Erlandsen method. The remaining 128 cases included 92 of incipient pulmonary tuberculosis without softening, 5 of acute miliary tuberculosis and 31 cases chiefly presenting physical signs of chronic bronchitis and emphysema, asthma and bronchiectasis. In 11 of the last group, only Much granules were found. It is implied that the remainder either showed some bacilli or there were reasons why tubercular bacilli and degenerate forms did not appear in the sputum if any were present.

The technic was developed as follows: Modified Ellerman and Erlandsen method: 15-20 C. C. of sputum mixed with an equal quantity of 60% sodium carbonate solution in a stoppered flask and incubated at 37 deg. 24-48 hours; centrifuged; sediment mixed with equal volume of 30% antiformin, allowed to become homogeneous by standing 10-20 minutes; centrifuged half hour; second sediment stained. The stain consisted of 75% Ziehl-Neelsen carbol-fuchsin mixture and 25% of a solution prepared as follows: A saturated solution of methyl violet B. N. in alcohol is diluted 10 times with 2% phenol in water and filtered. The entire solution must be freshly prepared at least every 8 days and must be filtered before using, or the fuchsin will stain too feebly. (1). This mixed stain is used either hot as ordinarily for detecting tubercle bacilli or preferably at room temperature for 24-48 hours. (2) Lugol solution 5 minutes, heated in flame till steam rises. (3) 5% nitric acid 1 minute. (4) 3% HCl a few seconds. (5) Equal parts acetone and alcohol till no more color is given off. (6) Dry with filter paper. (7) Counterstain with Bismark brown 1 minute. (8) Wash in water. (9) Dry and mount. The Much granules appear black with a pale red halo. To avoid confusion with stain precipitation, cocci and fragments of cell nuclei, depend only on forms in rods or heaps of rods.

The paper contains a report on the addition of various dilutions of tubercle bacilli emulsions to non-tubercular sputum and the possibilities of thus detecting various quantitative infectious conditions. The Much granules are virulent to guinea pigs but may be destroyed or rendered non-virulent by the antiformin treatment, so that inoculations may fail on this account. Fat splitting enzymes derived from lymphocytes are probably responsible for the degeneration of bacilli into Much granules. As stated, cases with Much granules only, do not, unless very rarely, react to any modification of the tuberculin reaction, skin or conjunctival. Only special methods of search are able to detect the Much granule type.

